# NeuroPIpred: a tool to predict, design and scan insect neuropeptides

**DOI:** 10.1038/s41598-019-41538-x

**Published:** 2019-03-26

**Authors:** Piyush Agrawal, Sumit Kumar, Archana Singh, Gajendra P. S. Raghava, Indrakant K. Singh

**Affiliations:** 1 Department of Computational Biology, Indraprastha Institute of Information Technology, Okhla Phase 3, New Delhi, 110020 India; 2 Department of Bioinformatics, CSIR-Institute of Microbial Technology, Sector-39A, Chandigarh, 160036 India; 3 Molecular Biology Research Lab, Department of Zoology, Deshbandhu College, University of Delhi, New Delhi, 110019 India; 4 Department of Botany, Hans Raj College, University of Delhi, New Delhi, 110007 India

## Abstract

Insect neuropeptides and their associated receptors have been one of the potential targets for the pest control. The present study describes *in silico* models developed using natural and modified insect neuropeptides for predicting and designing new neuropeptides. Amino acid composition analysis revealed the preference of residues C, D, E, F, G, N, S, and Y in insect neuropeptides The positional residue preference analysis show that in natural neuropeptides residues like A, N, F, D, P, S, and I are preferred at N terminus and residues like L, R, P, F, N, and G are preferred at C terminus. Prediction models were developed using input features like amino acid and dipeptide composition, binary profiles and implementing different machine learning techniques. Dipeptide composition based SVM model performed best among all the models. In case of NeuroPIpred_DS1, model achieved an accuracy of 86.50% accuracy and 0.73 MCC on training dataset and 83.71% accuracy and 0.67 MCC on validation dataset whereas in case of NeuroPIpred_DS2, model achieved 97.47% accuracy and 0.95 MCC on training dataset and 97.93% accuracy and 0.96 MCC on validation dataset. In order to assist researchers, we created standalone and user friendly web server NeuroPIpred, available at (https://webs.iiitd.edu.in/raghava/neuropipred.)

## Introduction

**Figure 1:**
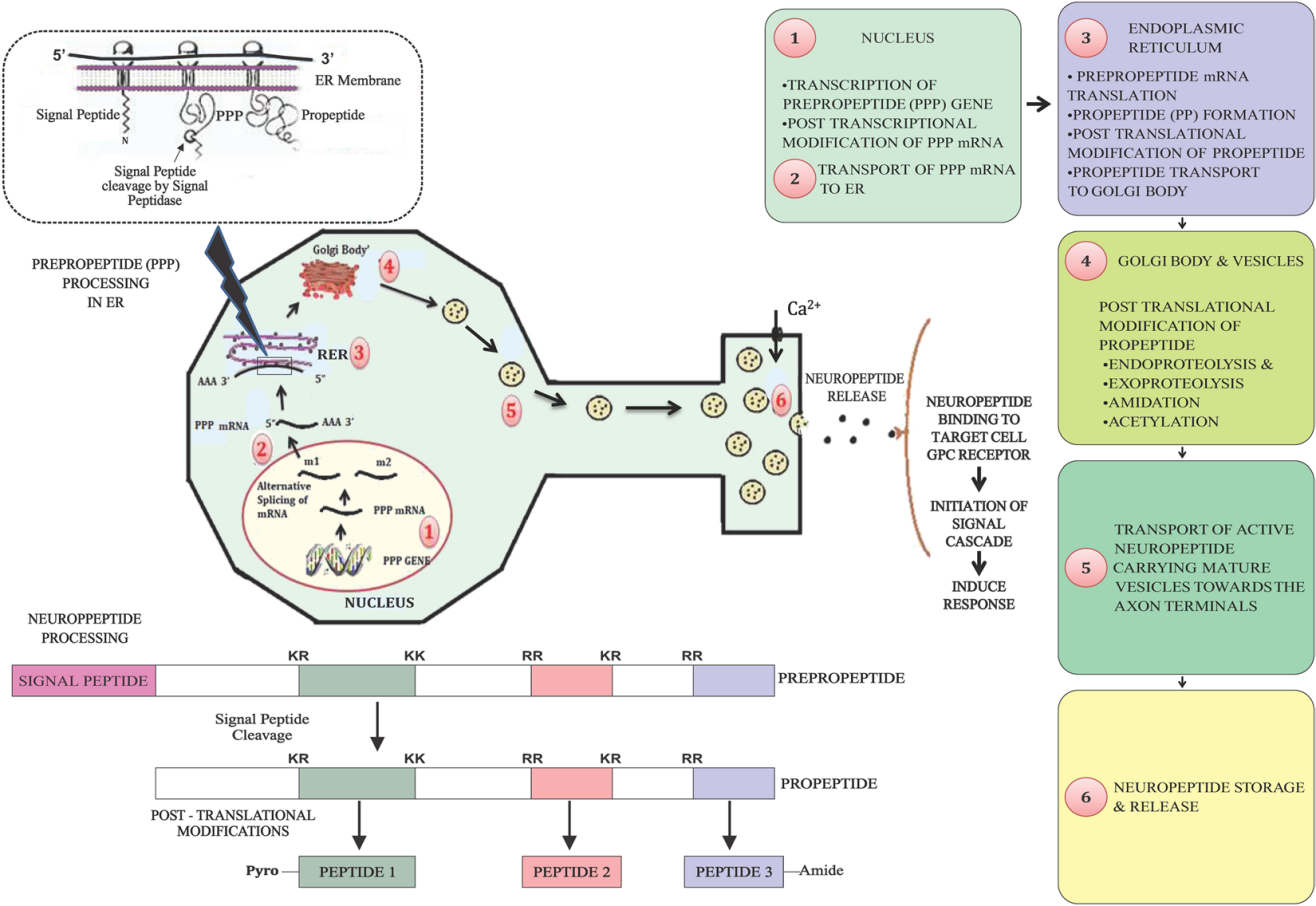
Schematic representation of insect neuropeptide biosynthesis and secretion.

Neuropeptides are one of the most versatile groups of neurotransmitter/neuromodulator secreted by central nervous system, which regulates various behavioural and physiological activities^1,2^. Neuropeptides are small peptides of around 5–80 amino acids^1^. Earliest report on neuropeptides was proposed by Stefan Kopec, a Polish scientist, in year 1922. Almost after 50 years two insect neuropeptides- proctolin and adipokinetic hormone were reported^3^. Neuropeptides are omnipresent in living organism, ranging from lower organism such as Cnidarians to complex ones like Bilaterians (including mammals)^4^. Neuropeptide genes have greatly evolved during the insect evolution^5^. Neuropeptides have been categorized into different groups according to their function such as myotropins, diuretic, AKH/RPCH family, eclosion hormone, pheromone biosynthesis activating peptides, allatotropins, allostatins, ecdysteroidgenesis, oostatic hormones. These neuromodulators are synthesized as precursor proteins known as prepropeptides in the neuronal cell body, which can produce one to numerous bioactive peptides via alternative splicing^6^. For instance in *Tribolium* two variants of diuretic hormone gene, DH37 and DH47 arise as a result of alternative splicing. Neuropeptide precursor (NPPs) undergoes several regulated cleavages to produce functionally active neuropeptides. Notably, a congregation of basic amino acids signifies these cleavage sites^7^. Thereby multiple copies of neuropeptides with different functional fates are produced. After post-translational modifications these mature neuropeptides are stored in synaptic vesicles near the axon terminals until neuronal stimulation. Upon neuronal stimulation i.e. axon terminal depolarization and Ca2^+^ influx these chemical modulators of neuronal circuits are released. Complete process of neuropeptide generation has been explained in Fig. 1.

Neuropeptides mainly binds to G-protein coupled receptors (GPCRs), a cell surface receptor that activates a cascade of reactions^8^. Neuropeptide-receptor complex initiates conformational changes in the receptor followed by downstream signaling within the target cell. NPs modulate variety of vital biological tasks. Brain NPs also targets different peripheral organs and regulates various behavioural and physiological activities. For instance in insects, it triggers various behavioural incidents like mating, migration and oviposition^1,2^. It also co-ordinates complex homeostatic activities such as metabolism, growth, development, water and ionic homeostasis^9^. Any abnormality in neuropeptides expression and regulation can lead to variety of severe neurological disorders. Detailed insight of neuropeptides i.e. its structure, function, mechanism of action and identification of putative neuropeptides will be useful in manipulating different biological systems including insects.

Extensive discoveries have been accomplished relating to insect neuropeptide identification and characterization in the recent past. In order to amalgamate this comprehensive data for its effective usage by scientific community various web resources have been designed such as NeuropPedia, NeuroPep and DINeR^10–12^. DINeR is a primary data source for insect neuropeptides wherein it gives details of sequence, functions, and receptor binding sites of the neuropeptides^12^. Neuropeptide’ is one of the database, which enlists information on gene families especially for vertebrate neuropeptides^13^. ‘NeuroPedia’ is another database, which provides information about neuropeptide sequence and its mass spectra libraries not only for insects but also of humans and other mammals. The information can be easily downloadable from this database, however, it does not cover arthropods class information^10^. ‘Neuropep’ is another database which maintains neuropeptide information of about 5949 peptides obtained from 493 organism belonging to 65 neuropeptide families. This database also maintains information about neuropeptides obtained from humans^11^. In addition, there are few prediction methods, which predict the cleavage site in the prepropetide, which may lead to potential neuropeptides. NeuroPID is one such machine learning based method, which predicts the neuropeptide precursors from the metazoan proteome^4^. NeuroPred is an another tool which predicts the cleavage site in the neuropeptide precursors and provides the peptide mass^14^. Recently, another tool NeuroPP has been published which utilizes compositional features (single, dipeptide and tripeptide) to predict neuropeptide precursors^15^. Further, it would be highly interesting to develop a tool that can directly predict the insect neuropeptide using features extracted from the already reported experimentally validated insect neuropeptide.

In order to complement previous studies, herein, we have made a systematic attempt to create a tool, which can predict the insect neuropeptides and provides structure and physicochemical properties of neuropeptides, using various machine-learning techniques. These machine-learning models are trained on various features extracted from the reported experimentally validated neuropeptides in the literature. In this study, we have pooled mass spectrometry data of insect neuropeptides form DINeR database into two datasets. These two datasets have been further divided into two, positive and negative datasets. Known neuropeptides constitute the ‘positive’ set, whereas the ‘negative’ sequences have functions unrelated to neuropeptides. Since negative peptides were not experimentally validated, we created negative dataset using SwissProt and SATPDB^16^. The overall objective of this study is to develop a tool, which can discriminate between insect neuropeptides and non-neuropeptides with high accuracy and allow users to generate mutant analogs of neuropeptides, which can be potential neuropeptide-based insecticides.

## Results

### Residue composition analysis

**Figure 2:**
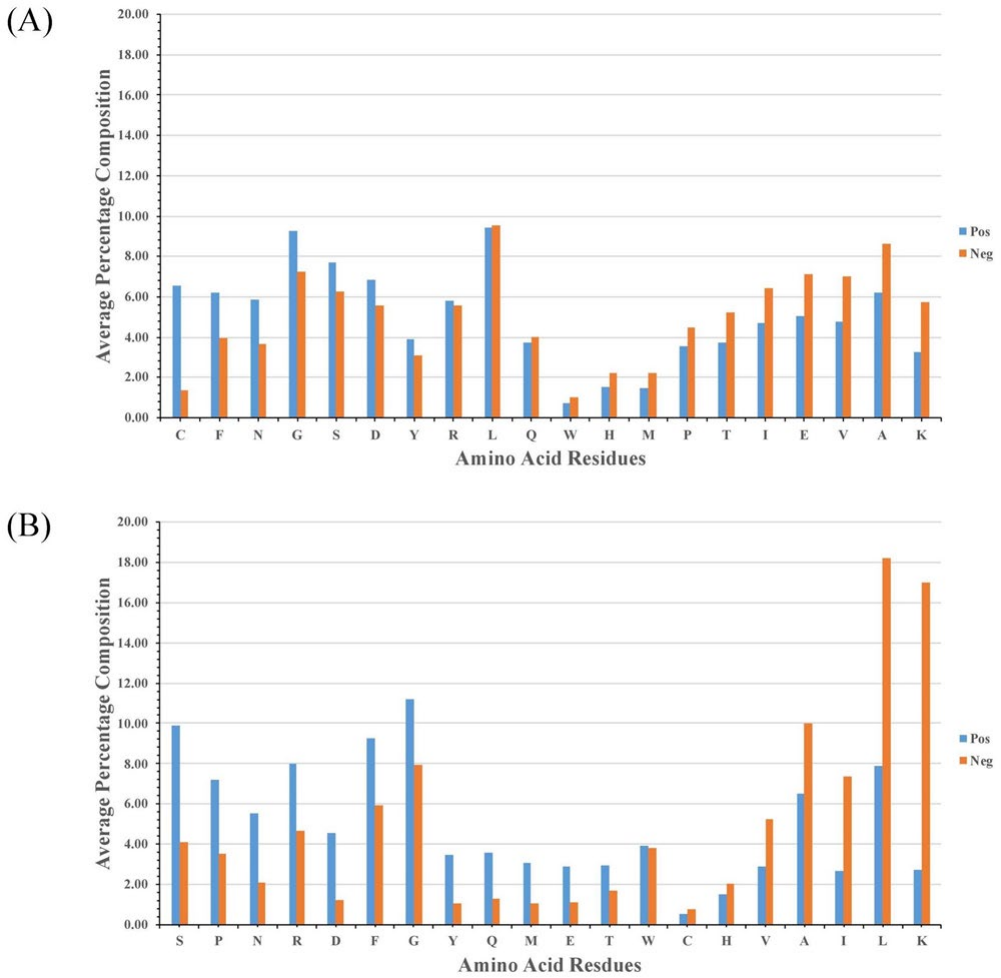
Comparison of percent average composition of residues present in (**A**) natural insect neuropeptides and random peptides, (**B**) modified insect neuropeptides and modified bioactive peptides taken from SATPDB.

It is important to analyse the nature of neuropeptides before developing *in silico* prediction models. As we know there are 20 natural amino acids present in a peptide/protein, it is important to analyse the frequency of an amino acids present in insect neuropeptides. Thus, we calculated and compared the percent average composition of each residue present in our dataset (positive and negative peptides). In case of NeuroPIpred_DS1, we observed the abundance of C, D, F, G, N, S, and Y residues in positive dataset (Fig. 2(A)) whereas in case of NeuroPIpred_DS2, residues like D, E, F, G, M, N, P, R, S, and Y were abundant in positive dataset (Fig. 2(B)). Similar kind of results have already been shown in previous study where authors have created a database of neuropeptides and showed the amino acid composition distribution in neuropeptides^11^.

### Positional residue preference in insect neuropeptides

We computed average composition for each residue at first five places for positive and negative dataset for both N and C terminus in both the datasets i.e. NeuroPIpred_DS1 and NeuroPIpred_DS2. In case of NeuroPIpred_DS1, residue N, F, D, S, and I were highly preferred at N terminus (Supplementary Table S1) and residue L, F, N, G, and L were preferred at C terminus (Supplementary Table S2) for the position number 1, 2, 3, 4, and 5 respectively. In case of NeuroPIpred_DS2, residue A was preferred at 1^st^ position, P at 2^nd^ position and S at 3^rd^,4^th^ and 5^th^ position at N terminus (Supplementary Table S3) whereas at C terminus, residue L was preferred at 1^st^ position, R at 2^nd^ position, P at 3^rd^ position and F at 4^th^ and 5^th^ position (Supplementary Table S4).

### Motif analysis

We extracted exclusive motifs which were present in insect neuropeptides using MERCI software. In case of NeuroPIpred_DS1, exclusive motifs predominant in positive dataset includes “ECC”, “QCK”, “FDEI”, “EIDR”. Complete list is provided in Supplementary Table S5. In case of NeuroPIpred_DS2, some of the exclusive motifs found in positive dataset are “GPR”, “SFGL”, “WFGP”, “YSF”. For complete list refer Supplementary Table S6.

### Machine learning technique performance on various input features

*In silico* identification and designing of novel molecules/therapeutics has been in trend in the last few decades. It allows biologists to screen potential molecules in low cost and lesser time. The prediction models which have been developed in the past utilize features from experimentally verified data. These features are important in functioning of the molecules. Some of the important features which are present in the therapeutic peptides includes their amino acid composition, dipeptide composition, terminus residue composition, order of the amino acid present in the peptide, binary profiles of the residue, residue physicochemical properties, motifs which are exclusively present in one group of molecules and many more. In the previous studies, these features have been used in order to develop machine learning models in order to predict and design novel therapeutic molecules^17–23^. In this study also, we used different machine learning techniques like SVM, RF, J48, SMO and NB for developing prediction models on different input features. The results are explained below.

### Amino acid composition based models

Various machine learning prediction models were developed using amino acid composition as an input feature which is the simplest and an important feature. This feature provides the information about the type of residues present in the peptide and responsible for its activity. In case of amino acid composition based prediction models, Random Forest achieved the maximum accuracy of 86.00%, with 0.72 MCC on training datasets and 84.00% accuracy with 0.68 MCC on validation dataset for NeuroPIpred_DS1 (Table 1). In case of NeuroPIpred_DS2, SVM model in comparison to other techniques, achieved the highest accuracy of 96.95% with 0.94 MCC on training dataset and 97.23% accuracy with 0.94 MCC on validation dataset (Table 2). SVM based models performance, developed for first 5, 10 and 15 residues from N and C terminus, and their combined form is summarized in the Supplementary Tables S7 and S8 for NeuroPIpred_DS1 and NeuroPIpred_DS2 respectively.

**Table 1:** The performance of amino acid composition based models developed using different machine learning techniques on NeuroPIpred_DS1.

Machine Learning Techniques (Parameters)	Main Dataset					Validation Dataset				
	Sen	Spc	Acc	MCC	AUROC	Sen	Spc	Acc	MCC	AUROC
SVM (g = 0.001, c = 2, j = 2)	88.14	83.43	85.79	0.72	0.92	85.71	82.29	84.00	0.68	0.90
Random Forest (Ntree = 20)	86.29	85.71	86.00	0.72	0.93	83.43	84.57	84.00	0.68	0.91
SMO (g = 0.001, c = 4)	84.29	84.86	84.57	0.69	0.85	80.57	83.43	82.00	0.64	0.82
J48 (c = 0.1, m = 10)	81.86	80.43	81.14	0.62	0.84	80.00	81.71	80.86	0.62	0.86
Naive Bayes (Default)	82.29	80.57	81.43	0.63	0.87	76.00	79.43	77.71	0.55	0.83

*Sen: Sensitivity, **Spc**: Specificity, **Acc**: Accuracy, **MCC**: Matthews Correlation Coefficient, **AUROC**: Area Under the Receiver Operating Characteristic curve.

**Table 2:** The performance of amino acid composition based models developed using different machine learning techniques on NeuroPIpred_DS2.

Machine Learning Techniques (Parameters)	Main Dataset					Validation Dataset				
	Sen	Spc	Acc	MCC	AUROC	Sen	Spc	Acc	MCC	AUROC
SVM (g = 0.005, c = 2, j = 2)	97.28	96.53	96.95	0.94	0.99	97.55	96.83	97.23	0.94	0.99
Random Forest (Ntree = 60)	97.52	95.26	96.53	0.93	0.99	97.06	95.56	96.40	0.93	0.98
SMO (g = 0.001, c = 5)	97.96	94.16	96.29	0.92	0.96	98.28	96.19	97.37	0.95	0.97
J48 (c = 0.4, m = 3)	91.96	90.84	91.47	0.83	0.93	93.87	93.97	93.91	0.88	0.94
Naive Bayes (Default)	90.10	89.42	89.80	0.79	0.94	89.22	91.11	90.04	0.80	0.95

^*^Sen: Sensitivity, **Spc:** Specificity, **Acc:** Accuracy, **MCC:** Matthews Correlation Coefficient, **AUROC:** Area Under the Receiver Operating Characteristic curve.

### Dipeptide composition based models

Dipeptide composition not only encapsulates the composition information but also provides the insight about the neighbouring residues present in the peptide and how they regulate the activity of the residues present next to each other. We utilize this feature also for developing prediction models. In case of NeuroPIpred_DS1, SVM based model performed best in comparison to other techniques, with accuracy of 86.50% and MCC of 0.73 on training datasets and 83.71% accuracy and 0.67 MCC on validation dataset (Table 3). Similarly, in NeuroPIpred_DS2, SVM model showed the highest accuracy of 97.47% and MCC of 0.95 on training dataset and accuracy of 97.93% and MCC of 0.96 on validation dataset (Table 4).

**Table 3:** The performance of dipeptide composition based models developed using different machine learning techniques on NeuroPIpred_DS1.

Machine Learning Techniques (Parameters)	Main Dataset					Validation Dataset				
	Sen	Spc	Acc	MCC	AUROC	Sen	Spc	Acc	MCC	AUROC
SVM (g = 0.001, c = 1, j = 4)	87.57	85.43	86.50	0.73	0.93	82.29	85.14	83.71	0.67	0.91
Random Forest (Ntree = 70)	90.29	82.00	86.14	0.73	0.94	86.86	69.14	78.00	0.57	0.89
SMO (g = 0.0005, c = 5)	84.57	86.86	85.71	0.71	0.86	79.43	88.00	83.71	0.68	0.84
J48 (c = 0.3, m = 4)	80.00	81.57	80.79	0.62	0.85	76.57	83.43	80.00	0.60	0.84
Naive Bayes (Default)	76.29	73.14	74.71	0.49	0.75	75.43	70.29	72.86	0.46	0.72

^*^Sen: Sensitivity, **Spc:** Specificity, **Acc:** Accuracy, **MCC:** Matthews Correlation Coefficient, **AUROC:** Area Under the Receiver Operating Characteristic curve.

**Table 4:** The performance of dipeptide composition based models developed using different machine learning techniques on NeuroPIpred_DS2.

Machine Learning Techniques (Parameters)	Main Dataset					Validation Dataset				
	Sen	Spc	Acc	MCC	AUROC	Sen	Spc	Acc	MCC	AUROC
SVM (g = 0.001, c = 1, j = 3)	97.96	96.84	97.47	0.95	0.99	98.28	97.46	97.93	0.96	0.99
Random Forest (Ntree = 70)	97.83	96.21	97.12	0.94	0.99	97.55	93.97	95.99	0.92	0.99
SMO (g = 0.0005, c = 5)	98.21	96.29	97.36	0.95	0.97	98.28	96.51	97.51	0.95	0.97
J48 (c = 0.4, m = 3)	93.44	90.06	91.95	0.84	094	94.61	88.57	91.98	0.84	0.93
Naive Bayes (Default)	93.38	86.03	90.15	0.80	0.90	94.12	86.03	90.59	0.81	0.90

^*^Sen: Sensitivity, **Spc:** Specificity, **Acc:** Accuracy, **MCC:** Matthews Correlation Coefficient, **AUROC:** Area Under the Receiver Operating Characteristic curve.

Here also, we developed SVM models using part of peptides (first 5, 10 and 15 residues) from N and C terminus as well as their combined form and reported the performance in the Supplementary Tables S9 and S10 for NeuroPIpred_DS1 and NeuroPIpred_DS2 respectively.

### Binary profile based models

Binary Profile has been found to be an important feature while developing prediction models. It not only provides the composition information of a residue but also tells about its order in the peptide. SVM based models were developed utilizing binary profile as an input feature for the first 5, 10 and 15 residues from N terminus and C terminus as well as their combined form (i.e. N5C5, N10C10 and N15C15). For the first dataset i.e. NeuroPIpred_DS1, N10C10 model achieved the maximum accuracy of 84.95% with 0.70 MCC on training dataset and 86.23% accuracy with 0.72 MCC on validation dataset (Table 5). Likewise, in NeuroPIpred_DS2, N10C10 model showed the highest accuracy of 97.97% and 0.96 MCC on training dataset and 98.02% accuracy and 0.96 MCC on validation dataset (Table 6).

**Table 5:** The performance of SVM based model developed on NeuroPIpred_DS1, where models were developed using binary profile of part of peptide.

Features (Parameters)	Main Dataset					Validation Dataset				
	Sen	Spc	Acc	MCC	AUROC	Sen	Spc	Acc	MCC	AUROC
N5 (g = 0.05, c = 3, j = 2)	76.97	76.86	76.91	0.54	0.83	72.41	74.29	73.35	0.47	0.80
N10 (g = 0.1, c = 1, j = 4)	83.31	79.18	81.26	0.63	0.90	83.64	84.62	84.13	0.68	0.91
N15 (g = 0.005, c = 2, j = 1)	81.78	79.92	80.77	0.62	0.88	82.05	77.04	79.37	0.59	0.88
C5 (g = 0.05, c = 8, j = 2)	75.68	73.80	74.75	0.49	0.82	74.71	78.86	76.79	0.54	0.83
C10 (g = 0.05, c = 4, j = 3)	79.88	77.05	78.48	0.57	0.87	80.61	78.70	79.64	0.59	0.90
C15 (g = 0.1, c = 2, j = 2)	77.68	77.63	77.65	0.55	0.86	81.20	80.00	80.56	0.61	0.89
N5C5 (g = 0.05, c = 3, j = 4)	82.98	79.18	81.10	0.62	0.89	81.03	79.43	80.23	0.60	0.89
N10C10 (g = 0.05, c = 1, j = 1)	84.35	85.56	84.95	0.70	0.93	87.27	85.21	86.23	0.72	0.94
N15C15 (g = 0.05, c = 1, j = 1)	84.74	84.70	84.72	0.69	0.92	86.32	85.19	85.71	0.71	0.93

^*^Sen: Sensitivity, **Spc:** Specificity, **Acc:** Accuracy, **MCC:** Matthews Correlation Coefficient, **AUROC:** Area Under the Receiver Operating Characteristic curve, **N5/N10/N15:** First 5/10/15 elements from N-terminal, **C5/C10/C15:** First 5/10/15 elements from C-terminal, **N5C5/N10C10/N15C15:** First 5/10/15 elements from N-terminal as well as from C-terminal joined together.

**Table 6:** The performance of SVM based model developed on NeuroPIpred_DS2, where models were developed using binary profile of part of peptide.

Features (Parameters)	Main Dataset					Validation Dataset				
	Sen	Spc	Acc	MCC	AUROC	Sen	Spc	Acc	MCC	AUROC
N5 (g = 0.5, c = 2, j = 1)	95.11	93.92	94.59	0.89	0.99	94.85	93.97	94.47	0.89	0.99
N10 (g = 0.5, c = 2, j = 1)	97.56	94.32	95.80	0.92	0.99	98.83	94.30	96.40	0.93	0.99
N15 (g = 0.1, c = 2, j = 1)	97.22	94.60	95.63	0.91	0.99	98.43	96.79	97.45	0.95	0.99
C5 (g = 1, c = 1, j = 2)	97.52	97.39	97.47	0.95	0.99	97.55	96.51	97.10	0.94	0.99
C10 (g = 0.1, c = 2, j = 2)	98.07	96.86	97.41	0.95	0.99	99.22	96.64	97.84	0.96	0.99
C15 (g = 0.1, c = 2, j = 1)	98.93	95.57	96.89	0.94	0.99	99.21	92.51	95.22	0.91	0.99
N5C5 (g = 0.05, c = 3, j = 2)	98.27	97.24	97.81	0.96	0.99	98.77	97.78	98.34	0.97	0.99
N10C10 (g = 0.1, c = 2, j = 1)	98.48	97.54	97.97	0.96	0.99	98.83	97.32	98.02	0.96	0.99
N15C15 (g = 0.005, c = 1, j = 3)	97.86	97.09	97.39	0.95	0.99	97.64	96.79	97.13	0.94	0.99

^*^Sen: Sensitivity, **Spc:** Specificity, **Acc:** Accuracy, **MCC:** Matthews Correlation Coefficient, **AUROC:** Area Under the Receiver Operating Characteristic curve, **N5/N10/N15:** First 5/10/15 elements from N-terminal, **C5/C10/C15:** First 5/10/15 elements from C-terminal, **N5C5/N10C10/N15C15:** First 5/10/15 elements from N-terminal as well as from C-terminal joined together.

### Performance on additional dataset

We also evaluated the performance of prediction models developed using different input features (composition and binary profiles) on additional dataset. In case of NeuroPIpred_DS1, performance of model developed using amino acid composition declined from 84.00% accuracy to 78.00% as compared to validation dataset. Among all the models, N10C10 binary profile based model performed best with accuracy of 90.86% and MCC of 0.82 (Table 7). However, in case of NeuroPIpred_DS2, we didn’t observe any declined in the amino acid composition based model and it performed equally well as it performed for validation dataset. In this dataset too, N10C10 binary model performed best with accuracy of 98.45% and 0.97 MCC.

**Table 7:** The performance of SVM based models developed using different features on additional dataset.

Features (Parameters)	NeuroPIpred_Similar Dataset				
	Sen	Spc	Acc	MCC	AUROC
Amino acid composition (NeuroPIpred_DS1) (g = 0.1, c = 9, j = 1)	85.71	70.29	78.00	0.57	0.85
Amino acid composition (NeuroPIpred_DS2) (g = 0.1, c = 9, j = 1)	97.55	97.06	97.30	0.95	0.99
Dipeptide composition (NeuroPIpred_DS1) (g = 0.1, c = 9, j = 1)	82.29	84.57	83.43	0.67	0.91
Dipeptide composition (NeuroPIpred_DS2) (g = 0.1, c = 9, j = 1)	98.28	96.32	97.30	0.95	0.99
N10C10 Binary profile (NeuroPIpred_DS1) (g = 0.1, c = 9, j = 1)	87.27	94.25	90.86	0.82	0.97
N10C10 Binary profile (NeuroPIpred_DS2) (g = 0.1, c = 9, j = 1)	98.83	98.19	98.45	0.97	0.99

^*^Sen: Sensitivity, **Spc:** Specificity, **Acc:** Accuracy, **MCC:** Matthews Correlation Coefficient, **AUROC:** Area Under the Receiver Operating Characteristic curve, **N10C10:** First 10 elements form N-terminus and C-terminus respectively.

### Comparison with the existing methods

We compared the performance of the existing method NeuroPID with our method. We observed that NeuroPID showed the sensitivity (Sen) of 100%, specificity (Spc) of 5.14%, accuracy (Acc) of 52.57% and MCC of 0.16 in case of NeuroPIpred_DS1 validation dataset. In contrast to that, NeuroPIpred showed Sen of 82.29%, Spc of 85.14%, Acc of 83.71% and MCC of 0.67 for the same dataset (Table 8). The comparison shows that NeuroPIpred is better in discriminating neuropeptides from non-neuropeptides with higher accuracy and balanced sensitivity and specificity.

**Table 8:** Comparison of NeuroPIpred with existing method NeuroPID on the NeuroPIpred_DS1 validation dataset.

Method	Performance of benchmarking dataset NeuroPIpred_DS1							
	TP	TN	FP	FN	Sen	Spc	Acc	MCC
NuroPID	175	9	166	0	100.00	5.16	52.57	0.16
NeuroPIpred	144	149	26	31	82.29	85.14	83.71	0.67

### Implementation of web server

In this study, we have developed a web server which can discriminate between insect neuropeptides and non-neuropeptides. Thus, in order to assist researchers, we have implemented our two best models trained on two different datasets in our web server “NeuroPIpred”. “Natural model” is developed using dipeptide composition since SVM based model performed best in comparison to other models. This model will help in discriminating insect neuropeptides with non-neuropeptides consisting of only natural residues and are not modified. Second model “Modified model” is also SVM based model developed using dipeptide composition and performed better than other models. This model will help in discriminating insect neuropeptides with non-neuropeptides which are C-terminally modified with amide group. The server consists majorly of five modules (i) Predict; (ii) Design; (iii) Protein Scan; (iv) BLAST; and (v) Download.

(i) **Predict:** This module allows user to enter the multiple peptide sequence in a FASTA format or upload a file containing the same. Selected model will provide the prediction score at chosen threshold cut-off value and predict the nature of the peptide. User can also calculate the various physiochemical properties of their peptides using this page.
(ii) **Design:** Design module of NeuroPIpred allows users to design the insect neuropeptides with enhanced activity by suggesting best mutation. In this module, user needs to submit the peptide sequence in single line (no FASTA format is required), and server will generate all the possible mutants of the peptide with single mutation. These mutant peptides will be used for predicting the neuropeptides or non-neuropeptides nature of the peptide using the models provided at the server. The result page will display the prediction score and nature of the mutant peptides at the selected threshold value. User can sort the table to get the peptide with highest prediction score. Finally, user can select the best mutant peptide and submit it further for generating its mutant with the prediction score. This module will be useful in structure activity studies as well as in the case where user can design neuropeptide of desired activity.
(iii) **Protein Scan:** In this module, user can generate overlapping patterns of the protein sequence by selecting the required window length. The generated overlapping patterns are then used for predicting the class of the peptides. This module will help user to discover regions in the proteins which could possibly be neuropeptide.
(iv) **BLAST:** This module will help user in finding experimentally validated neuropeptides having similar sequence and properties to its given query peptide.
(v) **Download:** This module allows user to download the datasets used in this study which could be helpful for developing method with better performance and benchmarking other methods.

NeuroPIpred is freely accessible at https://webs.iiitd.edu.in/raghava/neuropipred.

## Discussion and Conclusion

Insect neuropeptides are small molecules, responsible for most of the physiological activities of an insect such as diuresis, signaling processes, pheromone synthesis and muscle activities. These small peptides and their target receptors have been potent and promising targets for pest control and developing new insecticidal agents. Number of neuropeptide or neuropeptide mimetic based therapeutics have been discovered in the past few decades which have been widely used for controlling pest from infecting various crops. For example, PBAN, Pss-PT, Lem-PK, Lom-MT-I-IV, and many more^24–26^. Detailed information of these therapeutic peptides has been described excellently in various studies^3,27^. In spite of so much of advancements, there are certain problems associated with these neuropeptides, which prevents them from being a strong insect control agent, such as their half-life, poor solubility in organic and aqueous solution, instability in the environment, rapid degradation in the insect digestive tract^3^.

**Figure 3:**
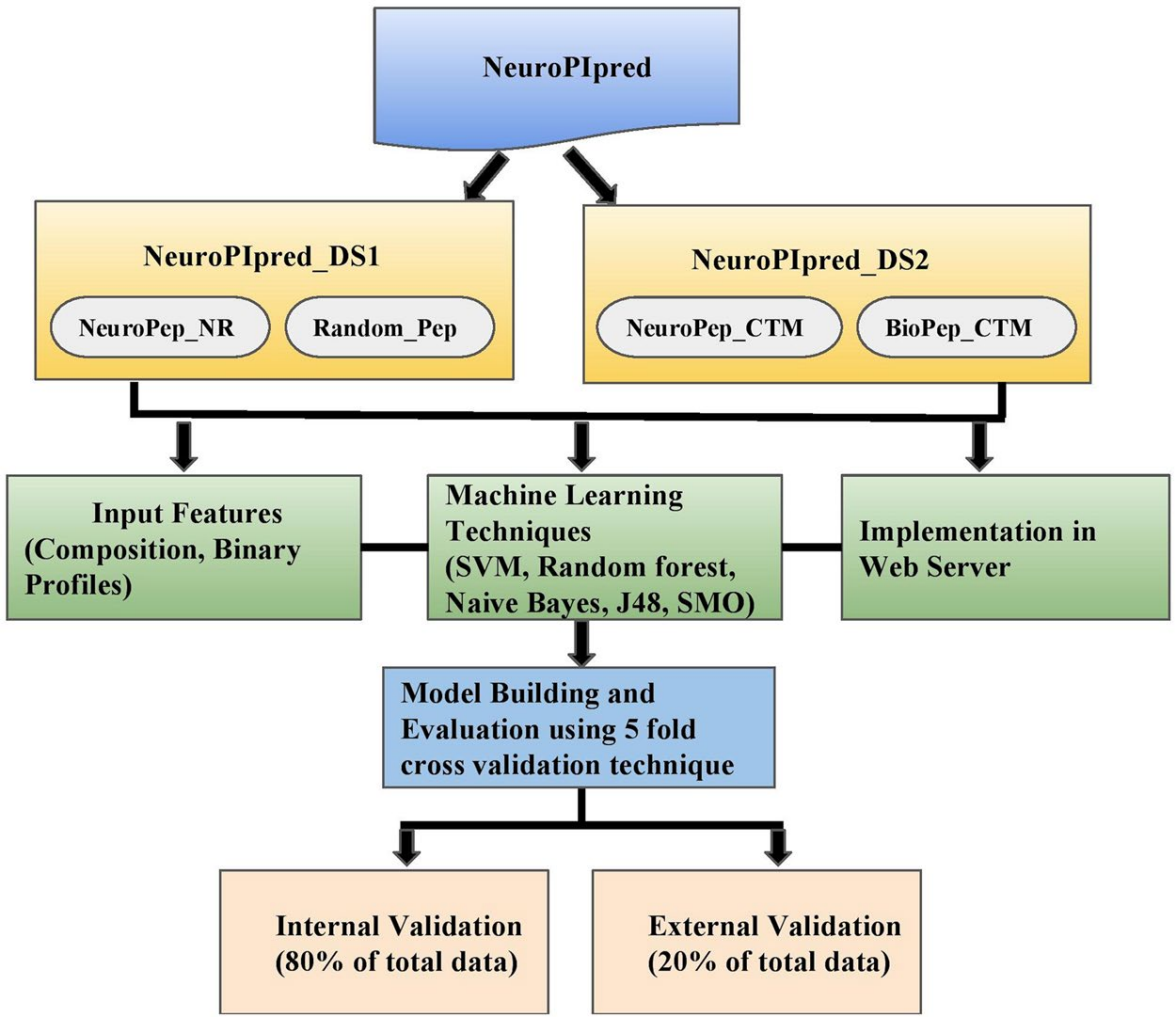
Schematic representation of workflow used for developing NeuroPIpred.

Currently to the best of authors knowledge, there is no method in the literature which can directly predict the nature of the peptide as neuropeptide or non-neuropeptide. Methods which have been designed are either for predicting neuropeptide precursors or for predicting neuropeptide cleavage sites in the protein. NeuroPIpred is the first attempt which allows user to predict the nature of the query peptide and make this method unique in comparison to other methods. Largest possible dataset was used for developing for the prediction models and both internal as well as external cross-validation techniques were used. In addition, we also developed method for predicting nature of chemically modified peptides. This method provides additional facility to users such as designing customized neuropeptides using Design module; or to find out the probable regions in the proteins which can be neuropeptide using its Protein Scan module. BLAST module allows user to check the similar peptides in the existing database of experimentally verified neuropeptides with its input peptides. In the present study, we have made a systemic attempt to predict and design novel neuropeptides with better insecticidal effects. We extracted the positive data from the DINeR database and negative data from SwissProt and SATPDB^16^. We created two data sets NeuroPIpred_DS1, which consists of natural neuropeptides and NeuroPIpred_DS2, which consists of C terminal amidated neuropeptides. Different input features were computed and machine-learning techniques were implemented using five-fold cross validation technique. Complete architecture of the algorithm is given in Fig. 3.

**Figure 4:**
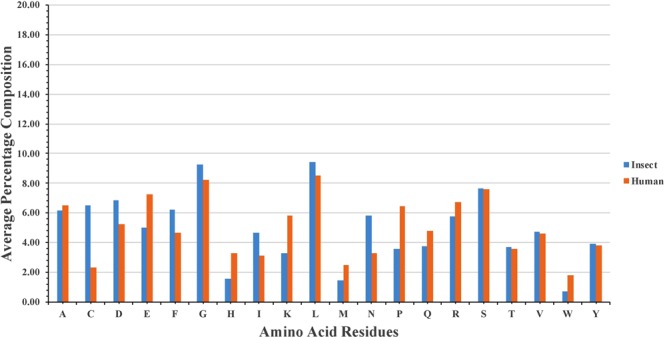
Comparison of percent average composition of residues present in insect neuropeptides and human neuropeptides.

Amino acid compositional analysis of the peptides revealed that insect neuropeptides are rich in C, D, F, G, N, S, and Y residues whereas C terminally amidated neuropeptides are rich in residues like D, E, F, G, M, N, P, R, S, and Y. We also compared the percent average composition of amino acid residues present in insect and human neuropeptides (extracted from NeuroPred and NeuroPID). We observed that insect neuropeptides are rich in residues like C, D, G, I, L and N whereas human neuropeptides are predominant in residues like E, H, K, M, P, Q and R (Fig. 4). This analysis shows that these two classes of neuropeptides are different from each other; hence, developing insect specific neuropeptide will not affect humans. Since there is no functional human neuropeptide prediction method available currently, we predicted the performance of our method by submitting human neuropeptides at our website and observed that our model predicted most of the human neuropeptide as non-neuropeptide. This proves that model developed in this study using insect neuropeptides can discriminate in between human and insect neuropeptides.

We also analysed the residue positional preference in the neuropeptides and found that in natural neuropeptides, residues N, F, D, S, and I are highly preferred at N terminus and residue L, F, N, G, and L are preferred at C terminus for the first five positions. In case of modified neuropeptides, we observed that residue A, P and S are preferred at N-terminus and residue L, R, P and F at C terminus. Motif analysis showed that motifs like ECC, QCK, FDEI, EIDR are predominant in natural neuropeptides and motifs like GPR, SFGL, WFGP, YSF in modified neuropeptides.

Various prediction models were developed using different features like composition (amino acid, dipeptide), binary profile and terminus composition. Different machine learning techniques like SVM light, Random Forest, J48, Naive Bayes and SMO were implemented for developing machine-learning models. Dipeptide composition based model outperformed all other models in case of both the datasets. An additional dataset where negative peptides are compositionally similar to positive peptides, was also created since discriminating similar peptides is a challenging task. We implemented machine-learning technique on this dataset also and observed that our N10C10 binary-based models was able to discriminate compositionally similar peptides with high accuracy. To assist scientific community, we have developed a web serve “NeuroPIpred” where we have implemented our best model. The server can be accessed from the link http://webs.iiitd.edu.in/raghava/neuropipred. Webserver is compatible to different media screens and can be used at either desktop, laptop, iPad or even on smartphones. Sometimes server don’t allows user to perform experiment on bulk of data. In order to address this problem, we have also provided the standalone of the method which allows user to perform experiment on large data. For standalone server, user needs to download the docker image “raghavagps/gpsrdocker” from the docker website.

## Methods

### Dataset creation

The amino acid sequences of neuropeptides were extracted from the recently developed database DINeR^12^, which comprises data of more than 50 neuropeptide families and over 400 different insect species. The database consists of around 4700 FASTA sequence of natural as well as modified neuropeptides. We created two different datasets NeuroPIpred_DS1 and NeuroPIpred_DS2, after removing peptides containing non-natural residues (BJOUZX) and repetitive sequences.

We removed repetitive sequences (or identical sequences which have 100% sequence identity) in order to remove biasness during model training. Thus, our dataset have unique sequences where no two sequences are identical. This is a commonly used practice in literature to avoid biasness in model training towards repetitive/identical sequences. However, if there was even a single residue difference between two sequences, we kept them in our study because in the previous studies it has been shown that even change in single residue will alter the peptide property^28,29^. This protocol is followed to avoid biasness while training the model. Motif and residue preference analysis was also performed on the unique dataset as similar type of sequences might favour certain type of residue which could not be true in reality.

Brief descriptions of these datasets are given below.

(i) **NeuroPIpred_DS1:** This dataset consists of 875 unique neuropeptides as positive dataset and for negative dataset; we randomly generated equal number of peptides from SwissProt since there is no repository where we can find experimentally validated non-neuropeptides. While generating random peptides from SwissProt, we made an assumption that the generated peptides do not possess neuropeptide property. It could be possible that the randomly generated peptide may have the neuropeptide activity, however the chances are very low. This approach is well established in cases, where experimentally validated negative data is not present^20,23,30^. The positive dataset was denoted as “NeuroPep_NR” and negative dataset as “Random_Pep”.
(ii) **NeuroPIpred_DS2:** This dataset consists of 2024 unique neuropeptides, having amide group at C terminus as modification. For negative dataset, we extracted peptides from SATPDB having same kind of modification which our positive peptides possess. SATPDB is a repository of peptides which consists of natural and modified peptides obtained from various peptide related database^16^. To create our negative dataset, we extracted peptides which possess the same modification as positive peptides; however they don’t demonstrates the same activity i.e. insect neuropeptide activity and may have any other activity. In total, we obtained 1582 such peptides after following the standard protocols. Here also, we termed positive dataset as “NeuroPep_CTM” whereas negative dataset as “BioPep_CTM”.

### Internal and external validation

The datasets were randomly divided into two parts. (i) Training dataset, which consists of 80% of total data, 700 positive and 700 negative peptides in case of NeuroPIpred_DS1, and 1616 positive and 1267 negative peptides in case of NeuroPIpred_DS2. (ii) Validation dataset, which consists of remaining 20% data, 175 positive and negative peptides in case of NeuroPIpred_DS1, and 408 positive and 315 negative peptides in case of NeuroPIpred_DS2.

In case of internal validation, prediction models were developed and evaluated using five-fold cross validation technique. In five-fold cross validation, sequences are divided randomly into five datasets, out of which any four datasets is used for training and remaining is used for testing. This process is repeated five times where each dataset is used at least one time for testing. Final result is calculated by averaging the performance of all five sets. In case of external validation, we evaluated the performance of the model developed using training dataset on validation dataset, which is very important for validating and evaluating any prediction method.

### Dataset for additional benchmarking

One of the biggest challenge while developing any prediction method is discriminating compositionally similar peptides with different activity^31,32^. We created two additional datasets “NeuroPIpred_Similar_DS1” and “NeuroPIpred_Similar_DS2” corresponding to NeuroPIpred_DS1 and NeuroPIpred_DS2 respectively. Similar approach was followed for creating the additional dataset, where positive peptide consists of neuropeptides and negative peptides are the peptides showing highest compositional similarity to positive peptides. Euclidean distance between two peptides composition were computed for identifying compositionally similar peptides and peptides with minimum Euclidean distance were selected. This type of approach has been followed in earlier studies^33,34^.

### Positional residues preference in insect neuropeptides

In order to know, which residue is preferred at which position, we calculated average composition of each residue for first five positions from both N and C terminus. We also computed the difference between the average composition of each residue between positive and negative peptides to observe the variation in their occurrence. In previous studies, scientists have shown the importance of this kind of study^17,18^.

### Motif analysis

MERCI software was used for analyzing the motifs uniquely present in neuropeptides. We used the default parameters for running the software^35^. Motif analysis provides the information related to different kind of patterns, which could be present in the neuropeptides.

### Input features for prediction

We used various input features and applied various machine learning techniques for developing prediction models. These features are described below.

**Figure 5:**
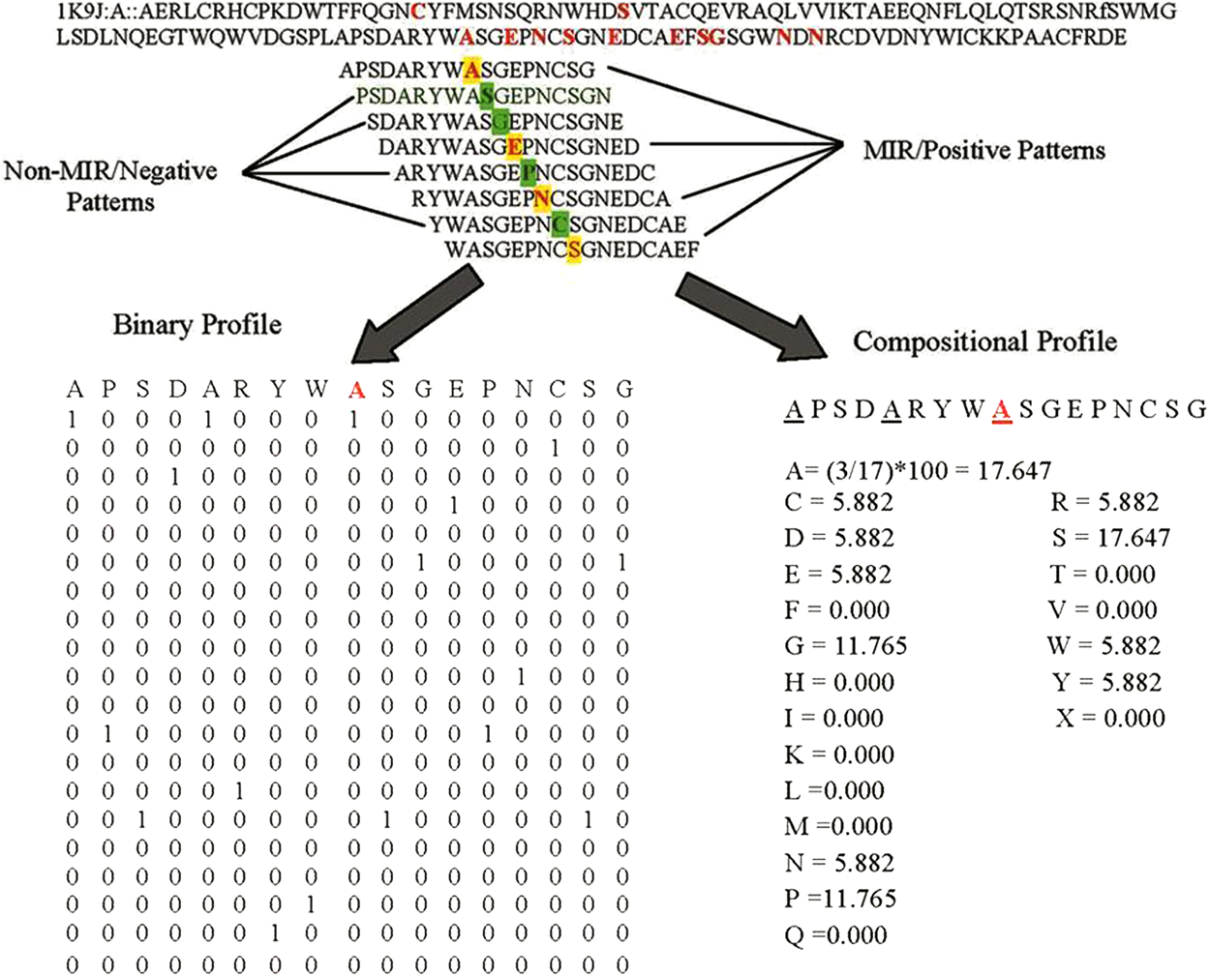
Schematic representation of generation of binary profiles. [Figure adapted from PLoS One 2011;6(9):e24039].

- **Amino acid composition:** Residue composition provides us insight about the fraction of amino acid type present within the peptide. In previous studies, amino acid composition has been used to classify two class of peptides using various machine learning techniques^29,36^. Equation 1 was used to calculate the composition of the peptide which provides a vector of dimension 20.
  1 $$Comp(i)=\frac{Ri}{N}\ast 100$$
  here, Comp (i) is the amino acid percent composition (i); R_i_ is the number of residues of type i, and N represents the total number of peptide’s residues.
- **Dipeptide composition:** Dipeptide composition is another type of input features which provided the information about the composition of pair of residues with the dimension of 400 (20 * 20). In order to calculate dipeptide composition, we count the occurrence of each type of dipeptide present in the given sequence and divide it by 400 which is total number of all possible dipeptides (AA, AC, AD………YV, YW, YY). Dipeptide composition provides the information regarding the fraction of amino acid as well as their local order. It is calculated using the Eq. 2.
  2 $$Dipeptide\,fraction(i)=\frac{Total\,number\,of\,Dipeptide(i)}{N-1}\ast 100$$
  where Dipeptide (i) is a type of dipeptide out of 400 dipeptides and N is the length of the peptide.
- **Split composition:** Here, we compute the amino acid and dipeptide composition for the first 5, 10 and 15 residues from the N and C terminus each. We also joined these terminus sequence like N5C5, N10C10 and N15C15 and compute the composition.
- **Binary Profiles:** In this study the length of neuropeptides and non-neuropeptides is variable, thus generating pattern of fixed length is difficult. To address this issue, we generated binary profile for each peptide to get numerical representation of amino acid sequence of peptides. This mean we need to represent each type of amino acid by a number. It has been shown in literature, that amino acid can be represented by a vector of dimension 20. For example amino acid ‘A’ can be represented by (1,0,0,0,0,0,0,0,0,0,0,0,0,0,0,0,0,0,0,0), ‘C’ can be represented by (0,1,0,0,0,0,0,0,0,0,0,0,0,0,0,0,0,0,0,0), …..….. and residue ‘Y’ can be represented by 0,0,0,0,0,0,0,0,0,0,0,0,0,0,0,0,0,0,0,1). This profile is unique for each residue where the presence of the particular residue is denoted by ‘1’ and the absence by ‘0’ (Fig. 5). This approach has been used earlier in many studies^21,37–39^. In this study, we generated binary profile for fist 5, 10 and 15 residues from N terminus as well as from C-terminus. Binary profiles were also generated for N5C5, N10C10 and N15C15 segment of the peptides.

### Machine learning techniques

Various machine learning techniques were implemented in this study. Brief description of these packages are provided below.

### Support vector machine (SVM)

SVM is one of the most successful machine learning technique used for classification and regression approaches^40–42^. In this study, SVM light Version 6.02^43^ of SVM was used for building prediction models. SVM light consists of various kernels for example linear, rbf, polynomial. Here, we used RBF kernel with various parameters; g € [10^−4^–10], c € [1–15], and j € [1–5]. RBF is a squared exponential kernel, which provides more functional space and flexibility than other kernels and hence gives better and optimum output. The classifier required input features of fixed length for training model, which could be employed for predicting values of unknown example.

### WEKA classifiers

WEKA is a complete package which provides number of machine learning classifier options for implementation^44^. We used 4 machine learning classifiers from this package namely Random Forest (RF), SMO, J48 and Naive Bayes (NB) in our study. We tuned different parameters present in these classifiers during run and reported the results obtained on the best parameters.

### Performance measure

We measured the performance of our methods using threshold dependent and threshold independent parameters. Threshold dependent parameters includes Sensitivity (Sen), Specificity (Spc), Accuracy (Acc) and Matthews Correlation Coefficient (MCC). These parameters are calculated using Eqs 3–6 as described below.

3 $$Sensitivity=\frac{TP}{TP+FN}\times 100$$

4 $$Specificity=\frac{TN}{TN+FP}\times 100$$

5 $$Accuracy=\frac{TP+TN}{TP+FP+TN+FN}\times 100$$

6 $$MCC=\frac{(TP\times TN)-(FP\times FN)}{\sqrt{(TP+FP)(TP+FN)(TN+FP)(TN+FN)}}$$

where TP represents correctly predicted positive value, TN represents the correctly predicted negative value, FP represents actual negative value which have been wrongly predicted as positive and FN represents positive value which have been wrongly predicted as negative.

In case of threshold independent parameter evaluation, Area Under Receiver Operating Characteristics (AUROC) curve was calculated where a ROC curve was drawn in between false positive and false negative rates.

### Comparison with the existing methods

We compared the performance of our method with the existing methods which have been designed to predict the neuropeptide precursors. We selected the recently developed software NeuroPID and evaluated the performance of the software on the independent dataset generated in this study. This dataset was selected because it has not been used in model training of both the software (NeuroPID and NeuroPIpred) hence results will not be biased. We submitted the sequence in the NeuroPID webserver and calculated the different performance measures.

## Supplementary information

Supplementary Information

## Data Availability

The dataset used in the study is freely available. User can download them from the Download section of the NeuroPIpred website.
