# Supplementary material for: NeuroPIpred: a tool to predict, design and scan insect neuropeptides
Source: Sci Rep. 2019 Mar 26;9:5129. doi: 10.1038/s41598-019-41538-x (PMC6435694; doi:10.1038/s41598-019-41538-x)
Supplement: Supplementary file 1 — Supplementary Information [file 41598_2019_41538_MOESM1_ESM.doc]

**NeuroPIpred: a tool to predict, design and scan insect neuropeptides**

**Piyush Agrawal^1,2^, Sumit Kumar^3^, Archana Singh^4^, Gajendra P. S. Raghava*^1^, Indrakant K. Singh*^3^**

1. Department of Computational Biology, Indraprastha Institute of Information Technology, Okhla Phase 3, New Delhi-110020, India.

2. Department of Bioinformatics, CSIR-Institute of Microbial Technology, Sector-39A, Chandigarh-160036, India.

3. Molecular Biology Research Lab, Department of Zoology, Deshbandhu College, University of Delhi, New Delhi-110019, India.

4. Department of Botany, Hans Raj College, University of Delhi, New Delhi-110007, India.

**Emails of Authors:**

PA: piyush_11@imtech.res.in

SK: [smtkmr388@gmail.com](mailto:smtkmr388@gmail.com)

AS: [archanasingh@hrc.du.ac.in](mailto:archanasingh@hrc.du.ac.in)

IKS: [iksingh@db.du.ac.in](mailto:iksingh@db.du.ac.in)

GPSR: raghava@iiitd.ac.in

**^*^ Equal Corresponding author**

1. Indrakant K. Singh, Assistant Professor, Department of Zoology, Deshbandhu College, University of Delhi, New Delhi 110019

E-mail address: iksingh@db.du.ac.in

Phone No: +91-11-26485525

2. Dr. G.P.S. Raghava, Professor, Centre for Computational Biology, IIIT Delhi, Okhla Industrial Estate, Phase III, New Delhi 110020

E-mail address: [raghava@iiitd.ac.in](mailto:raghava@iiitd.ac.in)

Phone No: +91-11-26907444

**Supplementary Information**

**Table S1. Positional preference of amino acids at N terminus of positive and negative peptides present in NeuroPIpred_DS1.**

| **Amino acid** | **Position1** | | | **Position2** | | | **Position3** | | | **Position4** | | | **Position5** | | |
| --- | --- | --- | --- | --- | --- | --- | --- | --- | --- | --- | --- | --- | --- | --- | --- |
|  | **Pos** | **Neg** | **Diff.**  **(Pos-Neg)** | **Pos** | **Neg** | **Diff.**  **(Pos-Neg)** | **Pos** | **Neg** | **Diff.**  **(Pos-Neg)** | **Pos** | **Neg** | **Diff.**  **(Pos-Neg)** | **Pos** | **Neg** | **Diff.**  **(Pos-Neg)** |
| **A** | 4.70 | 8.70 | -4.00 | 4.24 | 8.93 | -4.69 | 9.85 | 8.93 | 0.92 | 5.50 | 8.35 | -2.85 | 3.21 | 7.54 | -4.33 |
| **C** | 2.75 | 1.04 | 1.71 | 1.03 | 1.16 | -0.13 | 5.61 | 1.51 | 4.10 | 2.63 | 1.39 | 1.24 | 3.78 | 0.58 | 3.20 |
| **D** | 4.24 | 4.52 | -0.29 | 6.99 | 6.03 | 0.95 | 19.70 | 4.76 | 14.95 | 14.20 | 6.26 | 7.94 | 5.27 | 5.45 | -0.18 |
| **E** | 3.78 | 6.50 | -2.72 | 3.32 | 6.61 | -3.29 | 2.52 | 5.80 | -3.28 | 13.40 | 6.26 | 7.14 | 7.67 | 8.00 | -0.33 |
| **F** | 3.09 | 4.99 | -1.90 | 17.18 | 4.41 | 12.77 | 9.28 | 3.36 | 5.91 | 2.29 | 5.34 | -3.05 | 1.72 | 4.18 | -2.46 |
| **G** | 10.77 | 8.12 | 2.65 | 5.04 | 6.61 | -1.57 | 7.56 | 6.26 | 1.30 | 5.61 | 6.73 | -1.12 | 5.38 | 8.24 | -2.85 |
| **H** | 1.49 | 2.44 | -0.95 | 1.03 | 2.67 | -1.64 | 1.37 | 2.32 | -0.95 | 0.46 | 2.32 | -1.86 | 0.46 | 2.32 | -1.86 |
| **I** | 2.98 | 7.19 | -4.21 | 11.57 | 5.22 | 6.35 | 3.78 | 7.19 | -3.41 | 2.29 | 5.10 | -2.81 | 20.73 | 5.45 | 15.28 |
| **K** | 0.57 | 4.41 | -3.84 | 1.60 | 5.10 | -3.50 | 2.52 | 4.64 | -2.12 | 3.55 | 4.87 | -1.32 | 1.95 | 7.19 | -5.25 |
| **L** | 4.93 | 9.86 | -4.94 | 11.00 | 10.56 | 0.44 | 2.98 | 11.14 | -8.16 | 5.96 | 9.16 | -3.21 | 18.33 | 9.16 | 9.16 |
| **M** | 1.72 | 2.20 | -0.49 | 1.15 | 2.20 | -1.06 | 1.15 | 2.55 | -1.41 | 1.03 | 2.09 | -1.06 | 1.49 | 2.78 | -1.30 |
| **N** | 21.08 | 4.18 | 16.90 | 2.86 | 3.02 | -0.15 | 3.21 | 4.99 | -1.78 | 3.44 | 3.13 | 0.30 | 2.86 | 3.02 | -0.15 |
| **P** | 1.26 | 4.64 | -3.38 | 6.41 | 4.64 | 1.77 | 3.55 | 4.41 | -0.86 | 3.21 | 4.64 | -1.43 | 2.52 | 4.99 | -2.47 |
| **Q** | 7.45 | 3.48 | 3.97 | 5.04 | 4.29 | 0.75 | 3.55 | 3.13 | 0.42 | 3.78 | 3.83 | -0.05 | 3.21 | 3.13 | 0.08 |
| **R** | 1.37 | 5.80 | -4.43 | 4.12 | 5.68 | -1.56 | 2.98 | 4.87 | -1.89 | 2.86 | 5.68 | -2.82 | 4.35 | 6.03 | -1.68 |
| **S** | 19.24 | 5.57 | 13.68 | 3.32 | 5.80 | -2.48 | 6.87 | 7.08 | -0.20 | 17.18 | 5.80 | 11.38 | 8.13 | 5.57 | 2.56 |
| **T** | 1.95 | 6.03 | -4.09 | 1.60 | 5.45 | -3.85 | 2.52 | 5.57 | -3.05 | 6.07 | 5.80 | 0.27 | 1.72 | 4.76 | -3.04 |
| **V** | 2.29 | 7.19 | -4.90 | 9.39 | 6.84 | 2.55 | 7.45 | 6.96 | 0.49 | 3.78 | 8.47 | -4.69 | 4.24 | 7.54 | -3.30 |
| **W** | 0.80 | 0.93 | -0.13 | 0.80 | 1.16 | -0.36 | 0.92 | 1.39 | -0.48 | 0.69 | 1.62 | -0.94 | 0.92 | 0.81 | 0.10 |
| **Y** | 3.55 | 2.20 | 1.35 | 2.29 | 3.60 | -1.31 | 2.63 | 3.13 | -0.50 | 2.06 | 3.13 | -1.07 | 2.06 | 3.25 | -1.19 |

**Table S2. Positional preference of amino acids at C terminus of positive and negative peptides present in NeuroPIpred_DS1.**

| **Amino acid** | **Position1** | | | **Position2** | | | **Position3** | | | **Position4** | | | **Position5** | | |
| --- | --- | --- | --- | --- | --- | --- | --- | --- | --- | --- | --- | --- | --- | --- | --- |
|  | **Pos** | **Neg** | **Diff.**  **(Pos-Neg)** | **Pos** | **Neg** | **Diff.**  **(Pos-Neg)** | **Pos** | **Neg** | **Diff.**  **(Pos-Neg)** | **Pos** | **Neg** | **Diff.**  **(Pos-Neg)** | **Pos** | **Neg** | **Diff.**  **(Pos-Neg)** |
| **A** | 6.19 | 8.24 | -2.05 | 3.89 | 8.24 | -4.34 | 3.32 | 7.31 | -3.99 | 5.15 | 7.19 | -2.04 | 8.59 | 7.77 | 0.82 |
| **C** | 6.87 | 1.16 | 5.71 | 7.90 | 1.28 | 6.63 | 2.63 | 0.93 | 1.71 | 2.41 | 1.16 | 1.25 | 6.99 | 1.28 | 5.71 |
| **D** | 3.21 | 6.84 | -3.64 | 2.86 | 5.22 | -2.36 | 4.35 | 4.87 | -0.52 | 5.27 | 7.08 | -1.81 | 2.41 | 4.52 | -2.12 |
| **E** | 6.99 | 8.70 | -1.71 | 2.86 | 6.96 | -4.10 | 4.70 | 6.26 | -1.56 | 4.93 | 8.70 | -3.78 | 3.78 | 6.50 | -2.72 |
| **F** | 8.13 | 4.29 | 3.84 | 14.20 | 3.71 | 10.49 | 3.32 | 3.60 | -0.28 | 10.08 | 3.83 | 6.25 | 9.16 | 3.60 | 5.57 |
| **G** | 3.09 | 7.54 | -4.45 | 6.19 | 7.66 | -1.47 | 11.80 | 7.42 | 4.37 | 15.46 | 7.19 | 8.27 | 11.34 | 8.70 | 2.64 |
| **H** | 1.26 | 2.55 | -1.29 | 1.03 | 2.90 | -1.87 | 2.29 | 2.44 | -0.15 | 1.26 | 1.74 | -0.48 | 3.09 | 1.86 | 1.24 |
| **I** | 3.32 | 5.57 | -2.25 | 4.81 | 6.38 | -1.57 | 3.21 | 6.73 | -3.52 | 6.76 | 7.77 | -1.01 | 2.86 | 6.73 | -3.86 |
| **K** | 2.29 | 5.34 | -3.05 | 2.63 | 6.38 | -3.75 | 3.89 | 6.50 | -2.60 | 1.95 | 5.10 | -3.16 | 2.63 | 5.92 | -3.28 |
| **L** | 15.92 | 7.19 | 8.73 | 13.29 | 8.70 | 4.59 | 5.84 | 9.28 | -3.44 | 8.13 | 10.79 | -2.66 | 13.06 | 9.28 | 3.78 |
| **M** | 1.95 | 2.20 | -0.26 | 1.83 | 1.62 | 0.21 | 1.83 | 2.44 | -0.60 | 1.72 | 1.62 | 0.10 | 3.78 | 2.67 | 1.11 |
| **N** | 5.04 | 2.78 | 2.26 | 3.67 | 3.94 | -0.28 | 12.49 | 3.36 | 9.12 | 7.22 | 4.41 | 2.81 | 3.21 | 3.48 | -0.27 |
| **P** | 5.38 | 4.52 | 0.86 | 4.12 | 5.10 | -0.98 | 4.93 | 4.99 | -0.06 | 4.01 | 4.52 | -0.52 | 4.47 | 3.36 | 1.10 |
| **Q** | 5.84 | 4.76 | 1.09 | 3.89 | 4.52 | -0.63 | 3.67 | 3.94 | -0.28 | 1.72 | 3.60 | -1.88 | 1.37 | 5.34 | -3.96 |
| **R** | 3.21 | 5.68 | -2.48 | 4.70 | 5.57 | -0.87 | 5.61 | 5.92 | -0.30 | 4.01 | 5.34 | -1.33 | 5.61 | 5.34 | 0.28 |
| **S** | 5.04 | 6.61 | -1.57 | 4.12 | 6.38 | -2.26 | 12.37 | 6.26 | 6.11 | 6.76 | 5.22 | 1.54 | 5.27 | 6.50 | -1.23 |
| **T** | 3.44 | 5.34 | -1.90 | 2.98 | 5.22 | -2.24 | 6.30 | 5.34 | 0.96 | 1.83 | 4.52 | -2.69 | 4.35 | 5.45 | -1.10 |
| **V** | 8.71 | 7.42 | 1.28 | 5.96 | 6.84 | -0.89 | 3.89 | 8.58 | -4.69 | 7.10 | 6.50 | 0.61 | 2.98 | 7.42 | -4.45 |
| **W** | 0.34 | 0.46 | -0.12 | 0.23 | 1.39 | -1.16 | 0.11 | 1.04 | -0.93 | 0.57 | 0.58 | -0.01 | 1.49 | 1.28 | 0.21 |
| **Y** | 3.78 | 2.78 | 1.00 | 8.82 | 1.97 | 6.85 | 3.44 | 2.78 | 0.65 | 3.67 | 3.13 | 0.53 | 3.55 | 3.02 | 0.53 |

**Table S3. Positional preference of amino acids at N terminus of positive and negative peptides present in NeuroPIpred_DS2.**

| **Amino acid** | **Position1** | | | **Position2** | | | **Position3** | | | **Position4** | | | **Position5** | | |
| --- | --- | --- | --- | --- | --- | --- | --- | --- | --- | --- | --- | --- | --- | --- | --- |
|  | **Pos** | **Neg** | **Diff.**  **(Pos-Neg)** | **Pos** | **Neg** | **Diff.**  **(Pos-Neg)** | **Pos** | **Neg** | **Diff.**  **(Pos-Neg)** | **Pos** | **Neg** | **Diff.**  **(Pos-Neg)** | **Pos** | **Neg** | **Diff.**  **(Pos-Neg)** |
| **A** | 16.75 | 3.22 | 13.53 | 6.13 | 9.36 | -3.23 | 6.37 | 4.17 | 2.20 | 4.15 | 9.10 | -4.95 | 4.64 | 7.64 | -3.00 |
| **C** | 0.59 | 0.13 | 0.46 | 1.48 | 0.70 | 0.79 | 0.49 | 0.57 | -0.08 | 0.10 | 0.95 | -0.85 | 0.05 | 0.32 | -0.27 |
| **D** | 5.83 | 1.14 | 4.69 | 6.87 | 1.20 | 5.67 | 9.54 | 1.39 | 8.14 | 9.58 | 6.07 | 3.52 | 6.57 | 1.20 | 5.37 |
| **E** | 5.14 | 0.44 | 4.70 | 4.45 | 0.57 | 3.88 | 5.68 | 0.44 | 5.24 | 5.88 | 1.45 | 4.43 | 2.92 | 0.57 | 2.35 |
| **F** | 1.43 | 20.67 | -19.2 | 3.56 | 7.52 | -3.96 | 4.94 | 5.94 | -1.00 | 7.51 | 3.29 | 4.22 | 11.31 | 8.79 | 2.53 |
| **G** | 15.96 | 25.28 | -9.33 | 10.03 | 2.34 | 7.69 | 9.24 | 13.65 | -4.41 | 10.47 | 7.21 | 3.27 | 9.14 | 3.16 | 5.98 |
| **H** | 1.14 | 0.95 | 0.19 | 1.14 | 0.13 | 1.01 | 1.58 | 0.88 | 0.70 | 0.79 | 0.63 | 0.16 | 0.59 | 0.63 | -0.04 |
| **I** | 1.68 | 10.43 | -8.75 | 1.48 | 9.42 | -7.94 | 1.98 | 2.15 | -0.17 | 2.22 | 4.55 | -2.33 | 3.71 | 11.25 | -7.55 |
| **K** | 1.53 | 13.84 | -12.3 | 3.01 | 8.09 | -5.08 | 5.29 | 17.19 | -11.9 | 3.01 | 18.96 | -15.9 | 3.01 | 19.09 | -16.0 |
| **L** | 3.71 | 7.40 | -3.69 | 4.89 | 31.42 | -26.5 | 4.84 | 15.11 | -10.2 | 6.52 | 16.81 | -10.2 | 9.63 | 20.16 | -10.4 |
| **M** | 0.89 | 0.25 | 0.64 | 1.98 | 1.39 | 0.59 | 2.92 | 0.57 | 2.35 | 3.16 | 1.45 | 1.71 | 3.26 | 0.70 | 2.56 |
| **N** | 8.79 | 1.01 | 7.78 | 4.10 | 5.82 | -1.71 | 7.41 | 0.70 | 6.71 | 8.10 | 0.82 | 7.28 | 8.35 | 1.14 | 7.21 |
| **P** | 3.51 | 0.32 | 3.19 | 14.53 | 2.40 | 12.12 | 8.20 | 11.63 | -3.43 | 4.74 | 4.36 | 0.38 | 7.16 | 3.41 | 3.75 |
| **Q** | 5.43 | 2.53 | 2.91 | 4.84 | 1.14 | 3.70 | 5.04 | 0.95 | 4.09 | 3.95 | 0.76 | 3.19 | 3.26 | 0.57 | 2.69 |
| **R** | 2.72 | 4.93 | -2.21 | 3.46 | 3.79 | -0.33 | 3.90 | 4.17 | -0.27 | 4.55 | 5.12 | -0.57 | 2.27 | 3.73 | -1.46 |
| **S** | 16.35 | 1.83 | 14.52 | 13.44 | 1.71 | 11.73 | 13.24 | 4.11 | 9.13 | 11.46 | 8.72 | 2.74 | 12.06 | 3.16 | 8.90 |
| **T** | 5.63 | 0.25 | 5.38 | 2.82 | 0.32 | 2.50 | 2.96 | 0.95 | 2.02 | 4.05 | 2.47 | 1.59 | 2.62 | 3.98 | -1.36 |
| **V** | 1.98 | 3.48 | -1.50 | 2.57 | 3.67 | -1.10 | 2.92 | 1.96 | 0.96 | 2.12 | 1.52 | 0.61 | 2.67 | 8.22 | -5.55 |
| **W** | 0.30 | 1.07 | -0.78 | 6.42 | 8.72 | -2.30 | 1.38 | 12.26 | -10.8 | 1.88 | 4.87 | -2.99 | 1.48 | 1.64 | -0.16 |
| **Y** | 0.64 | 0.82 | -0.18 | 2.82 | 0.32 | 2.50 | 2.08 | 1.20 | 0.87 | 5.73 | 0.88 | 4.85 | 5.29 | 0.63 | 4.65 |

**Table S4. Positional preference of amino acids at C terminus of positive and negative peptides present in NeuroPIpred_DS2.**

| **Amino acid** | **Position1** | | | **Position2** | | | **Position3** | | | **Position4** | | | **Position5** | | |
| --- | --- | --- | --- | --- | --- | --- | --- | --- | --- | --- | --- | --- | --- | --- | --- |
|  | **Pos** | **Neg** | **Diff.**  **(Pos-Neg)** | **Pos** | **Neg** | **Diff.**  **(Pos-Neg)** | **Pos** | **Neg** | **Diff.**  **(Pos-Neg)** | **Pos** | **Neg** | **Diff.**  **(Pos-Neg)** | **Pos** | **Neg** | **Diff.**  **(Pos-Neg)** |
| **A** | 1.58 | 4.68 | -3.10 | 6.87 | 12.08 | -5.21 | 5.73 | 5.50 | 0.23 | 5.73 | 14.73 | -9.00 | 2.82 | 12.33 | -9.52 |
| **C** | 0.20 | 1.27 | -1.07 | 0.40 | 1.45 | -1.06 | 0.49 | 0.44 | 0.05 | 0.49 | 1.14 | -0.65 | 1.68 | 0.25 | 1.43 |
| **D** | 0.15 | 0.32 | -0.17 | 0.99 | 0.95 | 0.04 | 0.44 | 2.09 | -1.64 | 1.38 | 0.32 | 1.06 | 2.08 | 0.63 | 1.45 |
| **E** | 0.00 | 0.44 | -0.44 | 0.40 | 0.63 | -0.23 | 0.69 | 3.61 | -2.91 | 0.64 | 0.89 | -0.25 | 0.30 | 2.28 | -1.98 |
| **F** | 16.91 | 10.18 | 6.73 | 0.40 | 3.10 | -2.70 | 11.96 | 3.10 | 8.86 | 16.56 | 2.47 | 14.09 | 38.06 | 4.43 | 33.63 |
| **G** | 8.95 | 3.29 | 5.66 | 17.99 | 4.87 | 13.12 | 16.71 | 8.98 | 7.73 | 10.33 | 4.93 | 5.40 | 4.70 | 5.38 | -0.68 |
| **H** | 1.88 | 0.89 | 0.99 | 0.30 | 4.43 | -4.13 | 0.25 | 3.35 | -3.11 | 4.40 | 4.68 | -0.28 | 0.49 | 0.89 | -0.39 |
| **I** | 5.98 | 3.73 | 2.25 | 1.29 | 7.65 | -6.37 | 3.76 | 4.05 | -0.29 | 2.03 | 5.88 | -3.86 | 2.27 | 12.08 | -9.81 |
| **K** | 0.10 | 13.98 | -13.8 | 0.49 | 16.76 | -16.2 | 0.89 | 26.76 | -25.8 | 2.08 | 21.06 | -18.9 | 1.04 | 9.61 | -8.58 |
| **L** | 27.39 | 35.10 | -7.72 | 1.33 | 18.79 | -17.4 | 4.89 | 10.63 | -5.73 | 5.04 | 12.21 | -7.17 | 9.10 | 24.86 | -15.7 |
| **M** | 1.04 | 2.15 | -1.11 | 4.30 | 1.71 | 2.59 | 6.33 | 1.39 | 4.94 | 2.67 | 1.39 | 1.28 | 1.14 | 1.52 | -0.38 |
| **N** | 0.30 | 2.34 | -2.04 | 2.97 | 3.80 | -0.83 | 2.72 | 4.17 | -1.46 | 5.09 | 1.77 | 3.32 | 8.75 | 0.95 | 7.80 |
| **P** | 2.17 | 0.89 | 1.29 | 0.59 | 0.89 | -0.30 | 29.76 | 2.40 | 27.36 | 0.64 | 4.30 | -3.66 | 4.99 | 2.28 | 2.71 |
| **Q** | 0.25 | 2.59 | -2.35 | 0.35 | 1.83 | -1.49 | 0.94 | 1.45 | -0.51 | 3.86 | 0.89 | 2.97 | 0.59 | 0.76 | -0.17 |
| **R** | 12.85 | 7.78 | 5.07 | 43.50 | 7.21 | 36.29 | 2.03 | 4.43 | -2.40 | 6.92 | 3.29 | 3.63 | 0.89 | 4.49 | -3.60 |
| **S** | 0.30 | 3.10 | -2.80 | 5.09 | 2.85 | 2.25 | 9.59 | 5.95 | 3.64 | 15.57 | 7.91 | 7.66 | 4.10 | 3.61 | 0.50 |
| **T** | 0.54 | 0.51 | 0.03 | 1.93 | 1.45 | 0.47 | 1.24 | 2.09 | -0.85 | 3.71 | 4.43 | -0.72 | 2.67 | 0.70 | 1.97 |
| **V** | 5.83 | 4.62 | 1.21 | 2.42 | 6.01 | -3.59 | 1.24 | 4.36 | -3.13 | 3.61 | 3.98 | -0.38 | 1.33 | 8.92 | -7.58 |
| **W** | 9.79 | 1.20 | 8.59 | 8.06 | 1.77 | 6.29 | 0.05 | 2.97 | -2.92 | 5.34 | 2.97 | 2.37 | 0.20 | 2.91 | -2.71 |
| **Y** | 3.81 | 0.95 | 2.86 | 0.35 | 1.77 | -1.43 | 0.30 | 2.28 | -1.98 | 3.91 | 0.76 | 3.15 | 12.80 | 1.14 | 11.66 |

**Table S5. Exclusive motifs of NeuroPIpred_DS1.**

| **Sr. No.** | **Positive Motifs** | **Negative Motifs** |
| --- | --- | --- |
| 1 | ECC | KKL |
| 2 | QCK | FAA |
| 3 | FDEI | ITV |
| 4 | FDEID | TAL |
| 5 | NFDEI | EAI |
| 6 | NFDEID | TAA |
| 7 | EIDR | AFA |
| 8 | DEIDR | DIV |
| 9 | FDEIDR | IVA |
| 10 | NFDEIDR | KTA |

**Table S6. Exclusive motifs of NeuroPIpred_DS2.**

| **Sr. No.** | **Positive Motifs** | **Negative Motifs** |
| --- | --- | --- |
| 1 | GPR | AKK |
| 2 | FGPR | LAKK |
| 3 | SFGL | KWK |
| 4 | WFGP | FKK |
| 5 | WEGPR | FAK |
| 6 | YSF | KKF |
| 7 | YSFG | VGK |
| 8 | YSFGL | KKLL |
| 9 | GPRL | KLAK |
| 10 | FGPRL | LKKL |

**Table S7. The performance of SVM based models on NeuroPIpred_DS1, where models were developed using amino acid composition of part of peptides.**

| **Features**  **(Parameters)** | **Main Dataset** | | | | | **Validation Dataset** | | | | |
| --- | --- | --- | --- | --- | --- | --- | --- | --- | --- | --- |
|  | **Sen** | **Spc** | **Acc** | **MCC** | **AUROC** | **Sen** | **Spc** | **Acc** | **MCC** | **AUROC** |
| N5  (g=0.0001, c=7, j=2) | 76.68 | 73.80 | 75.25 | 0.51 | 0.80 | 70.69 | 75.43 | 73.07 | 0.46 | 0.77 |
| N10  (g=0.001, c=1, j=1) | 79.28 | 79.33 | 79.31 | 0.59 | 0.88 | 78.79 | 79.29 | 79.04 | 0.58 | 0.88 |
| N15  (g=0.001, c=2, j=2) | 83.37 | 83.75 | 83.58 | 0.67 | 0.89 | 80.34 | 76.30 | 78.17 | 0.56 | 0.88 |
| C5  (g=0.001, c=2, j=1) | 70.96 | 75.69 | 73.30 | 0.47 | 0.80 | 70.11 | 69.14 | 69.63 | 0.39 | 0.74 |
| C10  (g=0.001, c=1, j=2) | 77.94 | 74.62 | 76.30 | 0.53 | 0.84 | 75.15 | 74.56 | 74.85 | 0.50 | 0.80 |
| C15  (g=0.001, c=1, j=1) | 77.68 | 78.20 | 77.96 | 0.56 | 0.85 | 73.50 | 77.78 | 75.79 | 0.51 | 0.84 |
| N5C5  (g=0.0005, c=1, j=2) | 80.97 | 80.64 | 80.81 | 0.62 | 0.88 | 75.86 | 77.71 | 76.79 | 0.54 | 0.86 |
| N10C10  (g=0.0005, c=1, j=3) | 84.65 | 84.19 | 84.42 | 0.69 | 0.91 | 79.39 | 81.66 | 80.54 | 0.61 | 0.90 |
| N15C15  (g=0.001, c=3, j=1) | 87.24 | 82.60 | 84.72 | 0.70 | 0.91 | 82.91 | 79.26 | 80.95 | 0.62 | 0.90 |

*** Sen:** Sensitivity, **Spc:** Specificity, **Acc:** Accuracy, **MCC:** Matthews Correlation Coefficient, **AUROC:** Area Under the Receiver Operating Characteristic curve, **N5/N10/N15:** First 5/10/15 elements from N-terminal, **C5/C10/C15:** First 5/10/15 elements from C-terminal, **N5C5/N10C10/N15C15:** First 5/10/15 elements from N-terminal as well as from C-terminal joined together.

**Table S8. The performance of SVM based models on NeuroPIpred_DS2, where models were developed using amino acid composition** of part of peptides.

| **Features**  **(Parameters)** | **Main Dataset** | | | | | **Validation Dataset** | | | | |
| --- | --- | --- | --- | --- | --- | --- | --- | --- | --- | --- |
|  | **Sen** | **Spc** | **Acc** | **MCC** | **AUROC** | **Sen** | **Spc** | **Acc** | **MCC** | **AUROC** |
| N5  (g=0.0005, c=7, j=1) | 91.40 | 90.45 | 90.98 | 0.82 | 0.96 | 90.20 | 90.79 | 90.46 | 0.81 | 0.96 |
| N10  (g=0.001, c=2, j=5) | 95.53 | 94.15 | 94.78 | 0.90 | 0.98 | 95.33 | 94.63 | 94.95 | 0.90 | 0.98 |
| N15  (g=0.001, c=4, j=1) | 97.01 | 95.29 | 95.97 | 0.92 | 0.99 | 96.06 | 97.33 | 96.82 | 0.93 | 0.99 |
| C5  (g=0.001, c=2, j=2) | 95.67 | 94.15 | 95.00 | 0.90 | 0.98 | 93.87 | 97.14 | 95.30 | 0.91 | 0.99 |
| C10  (g=0.001, c=1, j=5) | 95.43 | 95.17 | 95.29 | 0.91 | 0.99 | 98.05 | 95.64 | 96.76 | 0.94 | 0.99 |
| C15  (g=0.005, c=2, j=1) | 98.72 | 96.12 | 97.14 | 0.94 | 0.99 | 100.00 | 96.79 | 98.09 | 0.96 | 1.00 |
| N5C5  (g=0.001, c=1, j=1) | 97.40 | 96.13 | 96.84 | 0.94 | 1.00 | 98.04 | 96.19 | 97.23 | 0.94 | 0.99 |
| N10C10  (g=0.0005, c=2, j=1) | 97.87 | 96.95 | 97.37 | 0.95 | 1.00 | 97.67 | 96.64 | 97.12 | 0.94 | 0.99 |
| N15C15  (g=0.0005, c=1, j=3) | 97.65 | 97.23 | 97.39 | 0.95 | 0.99 | 96.06 | 98.40 | 97.45 | 0.95 | 1.00 |

*** Sen:** Sensitivity, **Spc:** Specificity, **Acc:** Accuracy, **MCC:** Matthews Correlation Coefficient, **AUROC:** Area Under the Receiver Operating Characteristic curve, **N5/N10/N15:** First 5/10/15 elements from N-terminal, **C5/C10/C15:** First 5/10/15 elements from C-terminal, **N5C5/N10C10/N15C15:** First 5/10/15 elements from N-terminal as well as from C-terminal joined together.

**Table S9. The performance of SVM based models on NeuroPIpred_DS1, where models were developed using dipeptide composition** of part of peptides.

| **Features**  **(Parameters)** | **Main Dataset** | | | | | **Validation Dataset** | | | | |
| --- | --- | --- | --- | --- | --- | --- | --- | --- | --- | --- |
|  | **Sen** | **Spc** | **Acc** | **MCC** | **AUROC** | **Sen** | **Spc** | **Acc** | **MCC** | **AUROC** |
| N5  (g=0.0005, c=1, j=2) | 72.39 | 78.31 | 75.32 | 0.51 | 0.83 | 70.69 | 78.29 | 74.50 | 0.49 | 0.83 |
| N10  (g=0.001, c=1, j=1) | 80.18 | 84.65 | 82.39 | 0.65 | 0.90 | 78.18 | 87.57 | 82.93 | 0.66 | 0.90 |
| N15  (g=1e-05, c=10, j=2) | 83.14 | 80.31 | 81.60 | 0.63 | 0.89 | 77.78 | 82.22 | 80.16 | 0.60 | 0.87 |
| C5  (g=0.0005, c=1, j=1) | 73.82 | 73.22 | 73.52 | 0.47 | 0.81 | 78.74 | 73.71 | 76.22 | 0.53 | 0.82 |
| C10  (g=0.001, c=1, j=1) | 81.97 | 80.40 | 81.19 | 0.62 | 0.89 | 81.82 | 75.74 | 78.74 | 0.58 | 0.88 |
| C15  (g=5e-05, c=2, j=2) | 77.90 | 75.33 | 76.51 | 0.53 | 0.84 | 74.36 | 75.56 | 75.00 | 0.50 | 0.84 |
| N5C5  (g=0.0001, c=1, j=2) | 79.26 | 78.17 | 78.72 | 0.57 | 0.86 | 79.31 | 77.71 | 78.51 | 0.57 | 0.87 |
| N10C10  (g=1e-05, c=6, j=2) | 84.65 | 84.95 | 84.80 | 0.70 | 0.92 | 80.61 | 85.80 | 83.23 | 0.67 | 0.90 |
| N15C15  (g=0.001, c=1, j=1) | 85.42 | 83.37 | 84.30 | 0.69 | 0.92 | 81.20 | 87.41 | 84.52 | 0.69 | 0.91 |

*** Sen:** Sensitivity, **Spc:** Specificity, **Acc:** Accuracy, **MCC:** Matthews Correlation Coefficient, **AUROC:** Area Under the Receiver Operating Characteristic curve, **N5/N10/N15:** First 5/10/15 elements from N-terminal, **C5/C10/C15:** First 5/10/15 elements from C-terminal, **N5C5/N10C10/N15C15:** First 5/10/15 elements from N-terminal as well as from C-terminal joined together.

**Table S10. The performance of SVM based models on NeuroPIpred_DS2, where models were developed using dipeptide composition** of part of peptides.

| **Features**  **(Parameters)** | **Main Dataset** | | | | | **Validation Dataset** | | | | |
| --- | --- | --- | --- | --- | --- | --- | --- | --- | --- | --- |
|  | **Sen** | **Spc** | **Acc** | **MCC** | **AUROC** | **Sen** | **Spc** | **Acc** | **MCC** | **AUROC** |
| N5  (g=0.0005, c=1, j=2) | 94.80 | 91.00 | 93.13 | 0.86 | 0.97 | 94.85 | 92.38 | 93.78 | 0.87 | 0.98 |
| N10  (g=0.001, c=1, j=3) | 95.63 | 95.00 | 95.29 | 0.91 | 0.99 | 97.67 | 96.64 | 97.12 | 0.94 | 0.99 |
| N15  (g=0.001, c=1, j=1) | 96.79 | 96.40 | 96.55 | 0.93 | 0.99 | 96.85 | 97.33 | 97.13 | 0.94 | 1.00 |
| C5  (g=0.0001, c=2, j=2) | 96.10 | 94.55 | 95.42 | 0.91 | 0.99 | 96.32 | 94.92 | 95.71 | 0.91 | 0.99 |
| C10  (g=0.0005, c=1, j=3) | 97.16 | 96.53 | 96.81 | 0.94 | 0.99 | 98.05 | 95.97 | 96.94 | 0.94 | 0.99 |
| C15  (g=0.001, c=1, j=3) | 97.22 | 96.26 | 96.64 | 0.93 | 1.00 | 99.21 | 97.86 | 98.41 | 0.97 | 0.99 |
| N5C5  (g=0.0001, c=1, j=2) | 97.46 | 97.08 | 97.29 | 0.95 | 1.00 | 98.28 | 96.19 | 97.37 | 0.95 | 0.99 |
| N10C10  (g=0.0005, c=1, j=3) | 97.56 | 97.54 | 97.55 | 0.95 | 1.00 | 98.05 | 96.98 | 97.48 | 0.95 | 1.00 |
| N15C15  (g=0.0005, c=1, j=1) | 98.29 | 96.95 | 97.48 | 0.95 | 1.00 | 100.00 | 98.93 | 99.36 | 0.99 | 1.00 |

*** Sen:** Sensitivity, **Spc:** Specificity, **Acc:** Accuracy, **MCC:** Matthews Correlation Coefficient, **AUROC:** Area Under the Receiver Operating Characteristic curve, **N5/N10/N15:** First 5/10/15 elements from N-terminal, **C5/C10/C15:** First 5/10/15 elements from C-terminal, **N5C5/N10C10/N15C15:** First 5/10/15 elements from N-terminal as well as from C-terminal joined together.
